# Tumoral Stage of Mycosis Fungoides, Misdiagnosed With Wells Syndrome and Langerhans Cell Histiocytosis Histologically: A Challenging Case and Review of the Literature

**DOI:** 10.1002/cnr2.70412

**Published:** 2025-11-30

**Authors:** Seyed Mohammad Vahabi, Sahar Montazeri, Elnaz Pourgholi, Saeed Bahramian, Alireza Ghanadan, Kambiz Kamyab Hesari, Ifa Etesami

**Affiliations:** ^1^ Department of Dermatology, Razi Hospital, School of Medicine Tehran University of Medical Sciences Tehran Iran; ^2^ Department of Dermatopathology, Razi Hospital, School of Medicine Tehran University of Medical Sciences Tehran Iran; ^3^ Skin Research Center Shahid Beheshti University of Medical Sciences Tehran Iran

**Keywords:** cutaneous T‐cell lymphoma, eosinophilia, Langerhans cell histiocytosis, mycosis fungoides, tumor‐stage mycosis fungoides

## Abstract

**Background:**

Mycosis fungoides (MF) is a type of cutaneous T‐cell lymphoma (CTCL) with slow progression, usually presenting with patches and plaques. The infiltration of histiocytes and eosinophils in skin cancers can mask the underlying condition, posing a diagnostic challenge. While cases of MF associated with tissue eosinophilia or histiocytosis infiltration resembling Langerhans cell histiocytosis (LCH) have been reported, the simultaneous occurrence of both conditions in a single MF patient has not been documented.

**Case:**

In this case report, we presented an elderly man with a known history of patch and plaque‐stage MF who developed a large cutaneous tumor on his right mandibular margin. Multiple biopsies initially led to misdiagnoses, attributing the lesion to Well's syndrome and LCH due to the dense infiltration of eosinophils and histiocytes, respectively. However, the absence of a BRAF V600 mutation and lack of clinical correlation reduced the likelihood of these initial diagnoses. After repeated biopsies and a thorough evaluation by hematopathology and dermatopathology specialists, the findings were reassessed, leading to the consideration of tumoral MF with reactive eosinophil and histiocyte infiltrates. The patient was subsequently started on chemotherapy and phototherapy, which led to tumor regression after treatment initiation.

**Conclusion:**

This case highlights the importance of considering MF in patients presenting with tissue eosinophilia or Langerhans cell infiltration, even when initial biopsies lack definitive features.

## 1 Introduction

Mycosis fungoides (MF) is the most common form of cutaneous T‐cell lymphoma (CTCL), typically characterized by slow or non‐progressive disease that manifests as patches and plaques. The presentation of MF at the tumor stage as an initial sign is exceptionally rare [1, 2]. An abundant presence of Langerhans cells (LCs) and eosinophils can obscure the underlying CTCL, complicating diagnosis [3, 4]. While a limited number of MF cases with tissue eosinophilia (with or without peripheral blood eosinophilia) [5, 6, 7, 8, 9, 10] or dense infiltration of Langerhans cells [11, 12, 13] have been documented, the simultaneous infiltration of both cell types in a single MF patient has not been previously reported. Herein, we describe a diagnostically challenging case of tumoral MF with prominent reactive infiltration of LCs and eosinophils, which obscured the recognition of the underlying lymphoma.

## 2 Case Presentation

**FIGURE 1:**
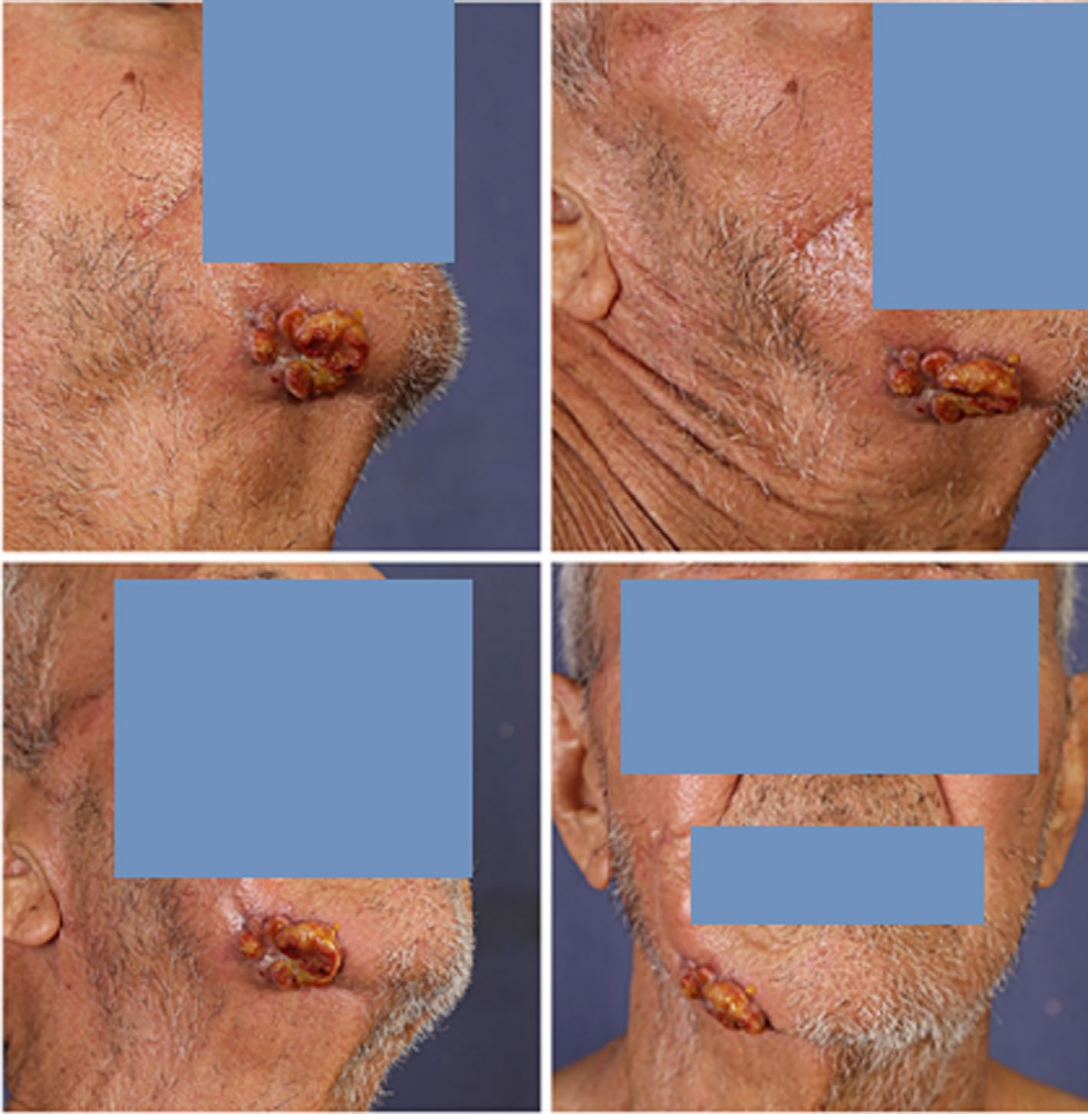
Clinical images of the patient with a tumoral lesion on the right mandibular margin. Right mandibular margin tumor measuring ~3 × 4 cm at presentation in May 2023.

**FIGURE 2:**
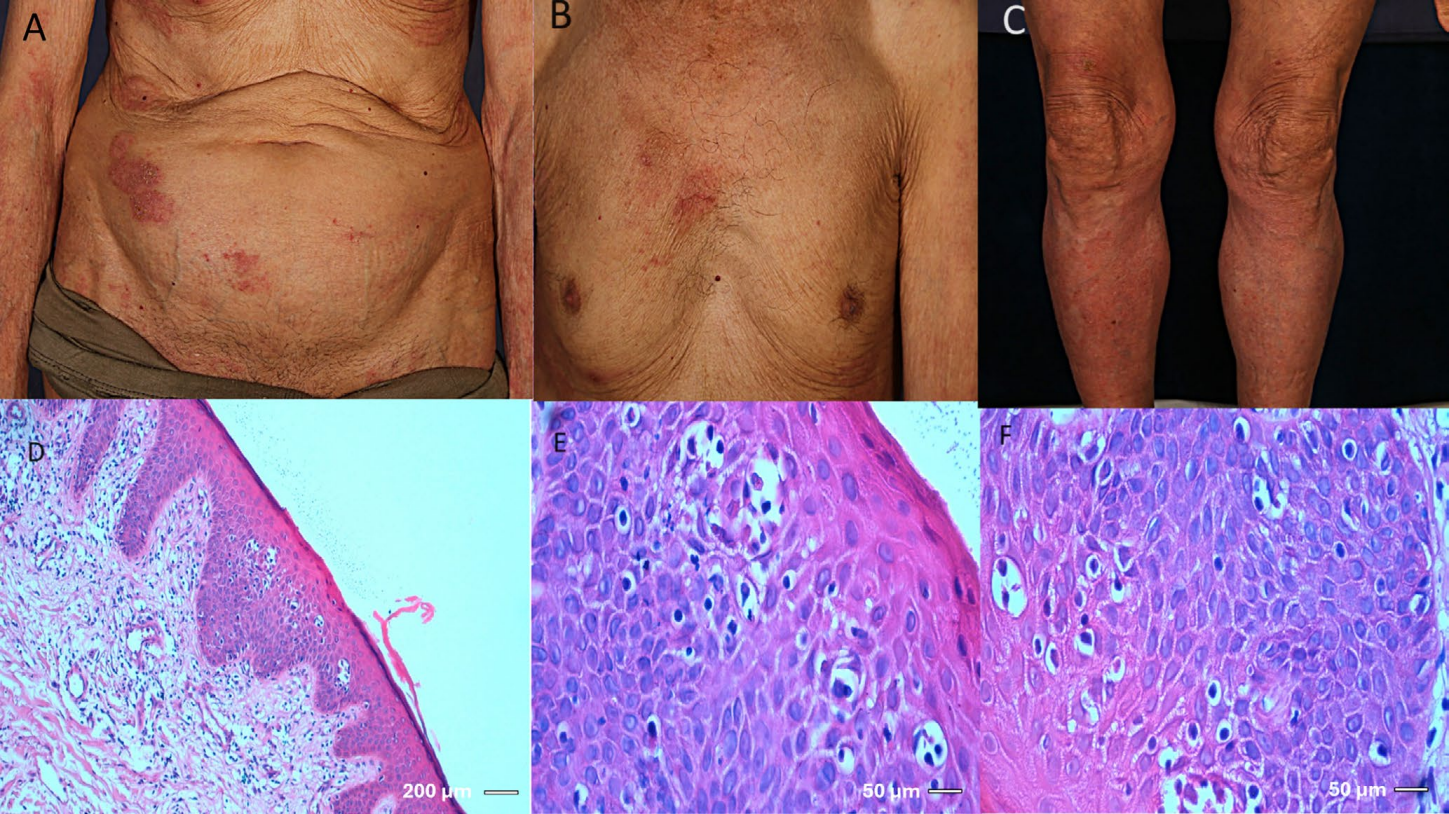
(A–C) Clinical images of patches/plaques. (D–F) H&E sections show acanthosis with epidermotropism of atypical lymphocytes. Stain: H&E. Magnification: (D) 100×; (E and F) 400×. Scale bars: (D) 200 μm; (E and F) 50 μm. Clinical pictures of previous MF (A–C). Epidermal acanthosis and epidermotropism of atypical lymphocytes are noted. Hematoxylin and eosin stain: (D) 100×, (E and F) 400×.

An 81‐year‐old man presented to the dermatology clinic at Razi Hospital, Tehran, Iran, in May 2023 with a rapidly growing cutaneous tumoral lesion, measuring 3 × 4 cm, on the right mandibular margin (Figure 1). The lesion had developed over the past 2 months. He had a 2‐year history of classic patch and plaque‐stage MF (Figure 2), showing partial improvement following narrow‐band ultraviolet B (NB‐UVB) phototherapy.

**FIGURE 3:**
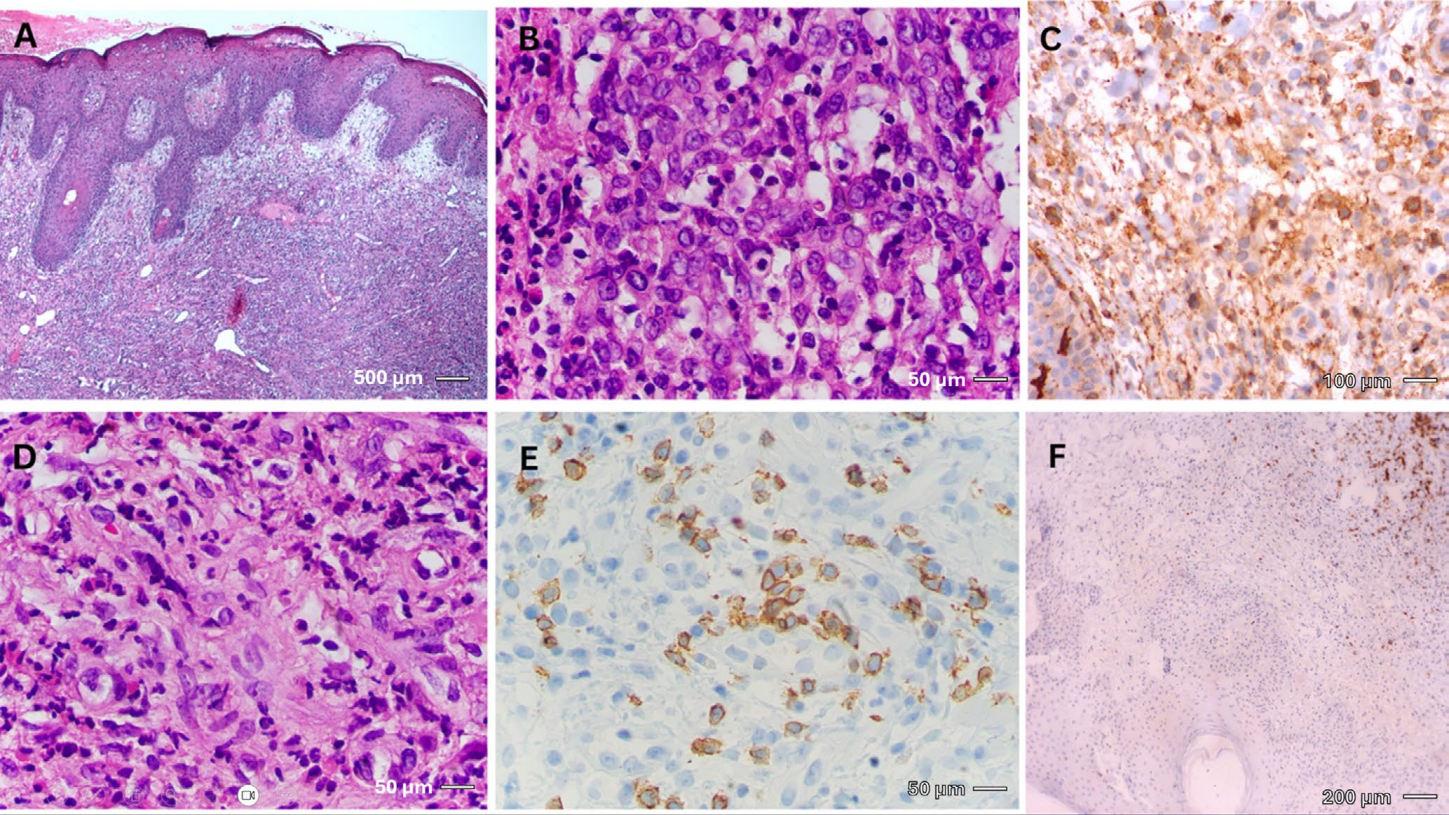
(A) Diffuse dermal inflammatory infiltrate. (B) Infiltrate composed of eosinophils admixed with large pale cells with vesicular nuclei. (C and D) CD1a immunostain highlights numerous Langerhans histiocytes. (E) CD3 highlights scattered T lymphocytes. (F) CD20 highlights rare B lymphocytes. Stains: H&E (A and B), CD1a (C and D), CD3 (E), CD20 (F). Magnification: (A) 40×; (B) 400×; (C) 100×; (D and E) 400×; (F) 100×. Scale bars: (A) 500 μm; (B) 50 μm; (C) 100 μm; (D and E) 50 μm; (F) 200 μm. BRAF V600 testing was negative on this biopsy. First biopsy: microscopic examination of biopsy sample taken from the enlarged chin lesion. (A) Dermis is diffusely infiltrated by inflammatory cells (hematoxylin and eosin stain, 40×). (B) The cellular infiltrate is mostly composed of large cells with pale eosinophilic cytoplasm and round to oval nuclei having a fine chromatin pattern and occasional small nucleoli (hematoxylin and eosin stain, 400×). (C and D) Immunohistochemistry staining using CD1a reveals that many large cells are Langerhans histiocytes (100× and 400×). (D) H&E stain (400×) showing a dense dermal inflammatory infiltrate. (E) Corresponding immunohistochemical stain for CD3 (400×) confirms the presence of numerous T lymphocytes within the infiltrate. The scattered distribution of CD3‐positive cells correlates with the lymphoid population seen in Panel D. (F) Immunostaining with CD20 shows few infiltrating B lymphocytes (100×).

The initial biopsy of the tumoral lesion revealed numerous large clear cells with vesicular nuclei, raising suspicion of large‐cell lymphoma (Figure 3A,B). However, immunohistochemical (IHC) staining demonstrated a dense dermal infiltration of leukocytes and eosinophils along with CD1a‐positive histiocytes (Figure 3C–F; Table 1), suggesting a possible diagnosis of Langerhans cell histiocytosis (LCH). To further investigate, polymerase chain reaction (PCR) and real‐time PCR were performed to detect the BRAF V600 mutation, a hallmark of LCH, but the results were negative, ruling out this diagnosis.

**TABLE 1:** Immunohistochemistry (IHC) staining was done using antibodies against markers.

Markers	The second biopsy and first IHC	The third biopsy and second IHC
CD20	Positive in a few scattered small lymphocytes	Positive in some small mature lymphocytes
CD3	Positive in some small lymphoid cells	Negative
CD4	Positive in some small lymphoid cells	Positive in scattered lymphocytes as well as some histiocytes
CD34	Negative in infiltrating cells	Highlights vascular channels, otherwise negative
CD30	Positive in a few medium to large lymphoid cells	Positive in scattered small mature lymphocytes
ALK	Negative	Not checked
CD1a	Positive in some Langerhans cells in the dermis	Negative
CD7	Not checked	Positive in rare scattered small mature lymphocytes
CD8	Not checked	Positive in rare scattered small mature lymphocytes
CD68	Not checked	Highlights scattered histiocytes
CD31	Not checked	Negative
Ki67	Not checked	Less than 5%
LCA	Not checked	Positive

**FIGURE 4:**
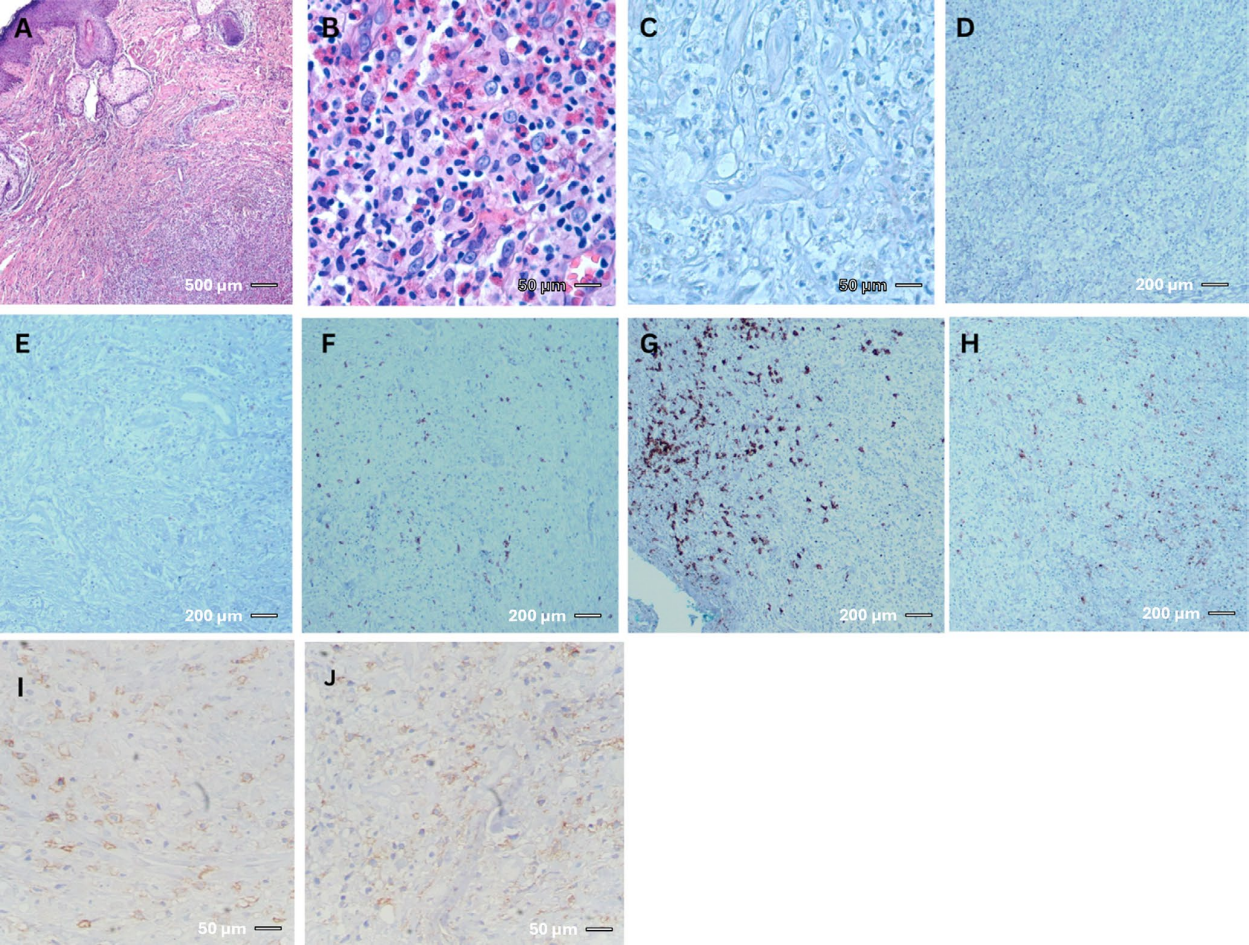
(A and B) H&E shows eosinophil‐rich inflammatory infiltrate. (C) CD1a negative. (D–F) T‐cell markers: CD3, CD4, CD8 highlight few/scattered T lymphocytes. (G) CD20 highlights some B cells. (H) CD68 highlights histiocytes. (I and J) CD45/LCA confirms leukocytic origin of the large atypical cells. Stains: H&E (A and B), CD1a (C), CD3/CD4/CD8 (D–F), CD20 (G), CD68 (H), CD45 (I and J). Magnification: (A) 40×; (B and C) 400×; (D–H) 100×; (I and J) 400×. Scale bars: (A) 500 μm; (B and C) 50 μm; (D–H) 200 μm; (I and J) 50 μm. Third biopsy: the same as the previous sample, there is an eosinophil‐rich inflammatory infiltrate through the dermis in this specimen (hematoxylin and eosin stain, (A) 40× and (B) 400×). (C) Immunostaining for CD1a is negative (400×). (D–F) Few cells show positive staining for T cell markers CD3, CD4, and CD8, respectively (100×). (G) Some B lymphocytes present in the infiltrate are highlighted by the CD20 marker (100×). (H) CD68 marker shows the presence of some histiocytes among the inflammatory cells (100×). (I and J) Immunohistochemistry for CD45 (LCA) confirms the leukocytic origin of the large, atypical cells (400×).

Given the diagnostic uncertainty and the progressively enlarging mandibular mass, a second biopsy was performed. Histological examination revealed a dense eosinophilic infiltrate suspicious for Wells syndrome. However, the clinical presentation of an enlarging mass and lack of flame figures in histopathology was inconsistent with Wells syndrome, necessitating a third biopsy from the same lesion a few weeks later. Pathologic evaluation of the third biopsy revealed ulcerated lesions with dense eosinophilic infiltration but without significant atypical lymphocytic proliferation (Figure 4A,B, Figure S1). IHC showed scattered CD4‐positive lymphocytes, a few CD8‐positive lymphocytes, and some CD68‐positive histiocytes, while CD1a and CD3 staining were negative (Figure 4C–H; Table 1). Furthermore, IHC for leukocyte common antigen (LCA/CD45) was performed. The large atypical cells were positive for LCA, CD45 (Figure 4I,J), confirming their lymphoid origin. Additionally, the patient's complete blood count showed no evidence of peripheral eosinophilia.

Following consultation with hematopathology and dermatopathology specialists, a diagnosis of tumoral MF with reactive infiltration of eosinophils and histiocytes was considered, based on the patient's history of MF, despite the absence of a prominent, unequivocal atypical lymphoid infiltrate on H&E morphology. To assess systemic involvement, an ultrasound examination of the cervical, axillary, and inguinal lymph nodes was performed, which revealed no lymphadenopathy. A doppler ultrasound of the carotid and vertebral arteries was unremarkable. A computed tomography (CT) scan of the chest, abdomen, and pelvis showed no significant abnormalities aside from a few small renal cysts, spinal lordosis, and mild prostate enlargement.

After a multidisciplinary consultation with an oncologist, chemotherapy with low‐dose gemcitabine was initiated alongside continued phototherapy for the patch and plaque lesions. Remarkably, after the fourth chemotherapy session, the tumor size had reduced by approximately 50%. Two months into treatment, the patient remained in good general condition and continued to receive weekly phototherapy sessions. The timeline of diagnostic and therapeutic events is summarized in Table 2.

**TABLE 2:** Timeline of diagnostic and therapeutic events.

Time point	Event
2021–2023	History of classic patch/plaque‐stage mycosis fungoides, partially controlled with NB‐UVB phototherapy
May 2023	Presentation with a rapidly growing 3 × 4 cm tumor on the right mandibular margin
June 2023	First biopsy: dense eosinophil and CD1a + histiocyte infiltrate. Initial misdiagnosis of Langerhans cell histiocytosis considered
July 2023	Second biopsy: dense eosinophilic infiltrate. Differential diagnosis of Wells syndrome is considered
August 2023	Third biopsy: dense eosinophils and CD68+ histiocytes. Scattered T cells with aberrant CD3 loss
September 2023	Multidisciplinary consensus: diagnosis of tumoral MF with reactive infiltrates
October 2023	Initiation of low‐dose gemcitabine chemotherapy and continued NB‐UVB phototherapy
December 2023	After the fourth chemotherapy session: ~50% reduction in tumor size

## 3 Discussion

This study presented an elderly patient with a known history of MF who developed a tumoral lesion on the mandibular margin. The dense eosinophilic and histiocytic infiltration led to multiple misdiagnoses, including Wells syndrome and LCH, before the correct diagnosis of tumoral MF was confirmed. To our knowledge, this is the first reported case of tumoral MF with extensive reactive infiltration of both eosinophils and histiocytes, which obscured atypical lymphocytes and significantly complicated the diagnostic process. Here, “obscured” indicates that the dense reactive infiltrate masked a sparse, atypical T‐cell population on H&E, which was ultimately confirmed by immunohistochemistry and clinical context.

Histologic images were captured using an Olympus BX53 light microscope equipped with an Olympus DP74 digital camera. Scale bars were generated in Fiji/ImageJ (v.1.54f, National Institutes of Health, Bethesda, MD, USA) using the calibrated pixel size for each objective lens (40×, 100×, and 400×), and their lengths are specified in every figure legend. Only uniform, linear adjustments to brightness and contrast were applied across entire images; no individual features were added, removed, or selectively enhanced. Figure layouts and panel lettering were assembled in Adobe Illustrator (v.28.0, Adobe Inc., San Jose, CA, USA).

MF, the most common subtype of CTCL, has unclear pathogenesis involving macrophages, T‐helper cells, dendritic cells (DCs), and CD8+ T lymphocytes. Diagnosis is based on clinical, pathological, and molecular findings. Early stages show epidermotropism, while the tumoral MF is characterized by deep dermal infiltration [1, 2].

LCs are immature DCs functioning as epidermal immune cells. LCH is a DC‐related disorder characterized by large histiocytes expressing S100, CD1a, and CD207 [14, 15, 16]. Both LCs and DCs are present in various inflammatory skin diseases and lymphomas, with DCs playing a role in CTCL progression [17, 18]. Additionally, LCH is linked to multiple malignancies and may develop before, after, or concurrently with them [19].

The infiltration of MF lesions by histiocytes or the coexistence of MF and LCH has been rarely reported in the literature, with only five cases reported in the literature to date [11, 12, 13, 20, 21]. Christie et al. [20] and Lin et al. [21] each reported a case with pathological findings consistent with MF and concurrent LC infiltration, both diagnosed and treated as MF. As summarized in Table 3, three additional cases were diagnosed with MF following an initial confirmed diagnosis of LCH [11, 12, 13]. BRAF mutation analysis was performed in two of these cases, with negative results in both [12, 13]. In our case, the first biopsy revealed CD1a‐positive histiocytes, while the third biopsy showed CD68‐positive histiocytes. The presence of different histiocyte subtypes within the tumor suggested a reactive phenomenon rather than a clonal proliferation of histiocytes. This reactive infiltration of histiocytes, which act as antigen‐presenting cells during MF progression [17], may mimic LCH and contribute to diagnostic challenges. Furthermore, the immunophenotypic shift observed between the biopsies—from scattered CD3+ T cells admixed with a reactive infiltrate to a population of larger cells with loss of CD3 expression—is highly suggestive of clonal progression and aberrant antigen loss, a recognized feature of tumor‐stage MF [22]. This evolution in the tumor's phenotype further complicated the histological picture and underscores the aggressive nature of the progression in this case.

**TABLE 3:** Reported cases of mycosis fungoides after a diagnosis of Langerhans cell histiocytosis or initially misdiagnosed as Langerhans cell histiocytosis.

Study, age, sex	Past medical history	First clinical presentation and biopsy	Last clinical presentation and biopsy	Final and time to diagnosis	Treatment and outcome
Two cases of mycosis fungoides after initial diagnosis of Langerhans cell histiocytosis					
Błażewicz et al. [11], 40s, female	NA	Diffuse edematous skin lesions and numerous nodules localized on the face IHC: CD1a+, S100–, CD2–, CD3–, CD68–, CD56–, thiaphorin‐1–, CD21–, granzyme B–, CD5– Diagnosed with LCH received PDZ, cladribine, vinblastine	Localized trunk papular lesions and peripheral blood T‐cell lymphoma with no response to previous treatment + Review of previous biopsies by an expert	FMF 2 years	Chemotherapy, PUVA, IFN a, alloHSCT GVHD happened and received PDZ, MTX, ECP, bexarotene, IFN a, radiotherapy Died because of septic shock 3 years later
Weyand et al. [12], 8, female	A history of intermittent hypopigmented patches	Lymphohistiocytic infiltrate with increased CD1a and S100‐positive Langerhans cells, consistent with LCH BRAF mutation testing was negative No clonal T‐cell population was identified With diagnosis of LCH received PDZ and MTX	Persistent hypopigmentation with papules recurred with discontinuation of therapy Atypical epidermotropic CD8+ T cells displayed loss of CD2 and diminished expression of CD7 T‐cell receptor gene rearrangement study identified a clonal T‐cell population	HMF NA	Topical triamcinolone Phototherapy Complete resolution
A case of tumoral mycosis fungoides after initial misdiagnosis/mimicking of Langerhans cell histiocytosis					
Thingujam et al. [13], 54, male	Diabetes, Aadenoid cystic carcinoma of maxillary sinus, dermatitis‐like lesions for 10 years	Progressively enlarging mass of the right little finger Composed predominantly of lymphocytes and histiocytoid cells. Lymphocytes showed mild atypia; the histiocytoid cells, forming multiple nodules, possessed ovoid to spindle‐shaped nuclei with grooving, infiltrating the dermis and subcutis. IHC was positive for CD3, CD20, S100, CD1a, Langerin, and BRAF V600E, Ki‐67 (40%–50%)	Multiple erythematous patches, plaques, and nodular skin lesions over the trunk and four extremities Dyshidrotic dermatitis with epidermotropism by atypical lymphocytes. Atypical lymphoid cells with mild nuclear irregularities were identified, which infiltrated the dermis and subcutis associated with many Langerhans, some eosinophils were seen IHC was positive for CD3, CD4, CD5, CD25, CD30, Ki‐67 (about 50%), and negative for CD7, CD8, CD20. Negative BRAF V600 and Cyclin D1	Tumoral MF NA	Radiotherapy Phototherapy Partial remission

Abbreviations: alloHSCT, allogeneic hematopoietic stem cell transplantation; ECP, extracorporeal photopheresis; FMF, folliculotropic mycosis fungoides; GVHD, graft‐versus‐host disease; HMF, hypopigmented mycosis fungoides; IFN a, interferon alfa; IHC, immunohistochemistry; LCH, Langerhans cell histiocytosis; MF, mycosis fungoid; MTX, methotrexate; PDZ, prednisolone; PUVA, psoralen plus ultraviolet‐A radiation.

Tissue eosinophils are uncommon in early‐stage MF, but they can become prevalent as the disease progresses to advanced‐stage tumors and folliculotropic variations [23, 24]. This may result from a shift from a T‐helper 1 to a T‐helper 2 immunophenotype in longstanding MF [25, 26, 27, 28]. Notably, blood and tissue eosinophilia at diagnosis have been identified as poor prognostic indicators [8].

Wells syndrome was one of the differential diagnoses considered in our case, given the dense eosinophilic infiltrate. This rare, chronic inflammatory dermatitis presents with sudden erythematous papules and nodules, often with vesicles or bullae [29]. A hallmark histopathologic feature of Wells syndrome is the presence of “flame figures”; however, these were not observed in our patient's pathology slides. Notably, their absence is not unusual in the acute phase of the disease [30]. Despite histopathologic similarities, the patient's overall clinical presentation was inconsistent with Wells syndrome, making this diagnosis unlikely.

Several prior reports have documented cases of MF with significant tissue eosinophilia (Table 4) [5, 6, 7, 8, 9, 10]. Similar to our case, most of these patients required multiple biopsies before a definitive diagnosis of MF was established, resulting in delays in initiating appropriate treatment. Unlike our patient, seven out of nine cases exhibited concurrent peripheral eosinophilia.

**TABLE 4:** Reported cases of mycosis fungoides with tissue eosinophilia.

Study, age, sex	Presentation past medical history (PMH) blood eosinophilia (BE)	First biopsy	Last biopsy	Final diagnosis and delay to diagnosis	Treatment and outcome
Palermo et al. [5], 61, female	Erythematous, scaly, and pruritic patches on legs and trunk PMH: none BE: yes	Presence of cerebriform Sezary cells in the dermis and Pautrier's microabscess, many eosinophils without any sign of abnormality	NA	MF 0	Prednisolone Remission
Ishibashi et al. [6], 36, male	Pigmented erythroderma with poikiloderma, nodular lesions on the trunk and arms, leonine facies PMH: 20‐year history of recurrent bacterial skin infections, alopecia totalis at Age 12 BE: yes	NA	Non‐epidermotropic dermal and subcutaneous sheet‐like infiltration of large atypical lymphoid cells was seen Small lymphocytes, plasma cells, and eosinophils were also found in this sheet‐like infiltration IHC: positive for CD3, CD4, CD25, CD45, CD30, CD8, CD20, CD79a, and negative for CD10, CD15, CD34, CD56, EMA, LMP‐1, EBNA‐2, ALK	FMF 2 years	Prednisolone Phototherapy Died of sepsis after 2 months
Terada [7], 80, male	Erythematous patch in the trunk PMH: prostatic adenocarcinoma BE: yes	Mild infiltrations of lymphocytes and eosinophils. No atypical cells or cerebriform cells were seen	The infiltration of atypical medium‐sized lymphocytes regarded as lymphoma cells and numerous mature eosinophils in the hair follicles. Atypical follicular epithelial cells, atypical cells in the deep dermis, and poorly differentiated SCC IHC: positive for vimentin, CD45, CD3, CD4, CD5, CD43, CD45, P53 in 23%, Ki‐67 in54%, Cyclin D1, and negative for CD10, CD20, CD21, CD23, CD79α, bcl‐2, CD56, CD57, CD138, CD15, κ‐chain, λ‐chain, CD30, CD117	MF + SCC 6 years	NA NA
Pearson et al. [8], 72, male	Malodorous ulcerations on the hands, nearly erythrodermic with ulcerated palmar plaques PMH: melanoma BE: yes	Dermal infiltration by numerous large atypical cells overlying a mixed inflammatory infiltrate with eosinophils IHC: positive for CD4 positive, and negative for CD3, CD7, CD8, CD20, and S100	Atypical lymphoid infiltrate with epidermotropism and numerous eosinophils T‐cell receptor γ chain gene rearrangement studies from biopsies demonstrated monoclonality	MF 2 years	Romidepsin Chemotherapy Died
65, male	Small nonhealing ulcer on the right palm PMH: NA BE: NA	Superficial ulceration, copious dermal eosinophils, and eosinophilic cytoplasmic inclusions within keratinocytes	Dense inflammatory infiltrate in the dermis with a prevalence of eosinophils and medium to large‐sized lymphocytes with nuclear pleomorphism and frequent mitoses IHC were positive for CD4, but negative for CD5, CD8, CD30, and CD56 T‐cell receptor γ chain gene rearrangement studies demonstrated monoclonality	MF 3 months	Alternative medicine options and lost to follow‐up Died
Jaque et al. [9], 56, female	Generalized infiltrated erythematous‐violaceous plaques and diffuse facial tumoral thickening with cystic areas, giving an overall leonine appearance PMH: 5‐year history of progressive skin rash BE: no	Dense heavy dermal lymphoeosinophilic infiltrate, with no evidence of atypical lymphocytes	Greatly diminished eosinophilic infiltrate with underlying findings are finally consistent with FMF	FMF 4 months	Low‐dose interferon Alitretinoin Local radiotherapy Improved
69, male	A 10‐year refractory pruritic nummular rash PMH: NA BE: yes	Spongiotic psoriasiform dermatitis with a lymphoeosinophilic infiltrate, without any atypical lymphocytes or epidermotropism	Lymphoid atypia with prominent epidermotropism and folliculotropism with positive clonality	MF 5 years	Corticosteroid Phototherapy Various systemic agents including low‐dose oral chlorambucil Improved under treatment
72, female	Widespread comedogenic rash for 6 years PMH: alopecia of scalp and eyebrows BE: yes	Follicular hyperkeratosis and dense mixed perifollicular inflammation with marked eosinophils	Atypical lymphocytic infiltrate	FMF 4 years	Hydroxyurea Imatinib, interferon‐alpha, oral alitretinoin, phototherapy NA
Saki et al. [10], 60, female	Generalized pruritic papules, plaques, and nodules with pinkish color multiple excoriation lines, and yellowish crusts PMH: prurigo nodularis for 3 years BE: yes	Hyperkeratosis, parakeratosis, acanthosis, spongiosis, vesicle formation, dense patchy infiltrates of mature as well as atypical lymphocytes intermingled with certain eosinophils in the dermis	Hyperkeratosis, parakeratosis, acanthosis with multifocal epidermotropism of atypical lymphocytes with enlarged hyperchromatic nuclei and irregular border mainly in the basal layer. Patchy dense infiltrates of essentially lymphoid cells with enlarged irregular nuclei admixed with some eosinophils in the dermis IHC: positive for CD3, CD4, CD5, CD8, Ki67 in more than 50% of the lymphoid population and negative for CD20, CD30, CD7	MF NA	Phototherapy, systemic acitretin Lost to follow‐up

Abbreviations: ALK, anaplastic lymphoma kinase; EBNA‐2, Epstein–Barr virus‐encoded nuclear antigen 2; EMA, epithelial membrane antigen; FMF, folliculotropic mycosis fungoides; IHC, immunohistochemistry; LMP‐1, latent membrane protein‐1; MF, mycosis fungoides; SCC, squamous cell carcinoma.

### 3.1 The Key Challenge

The overwhelming eosinophilic and histiocytic infiltrates masked the sparse atypical T‐cell population, causing multiple misdiagnoses and delaying treatment.

- Key finding: the final diagnosis was achieved by synthesizing (i) clinical history of MF, (ii) absence of BRAF V600E mutation (excluding LCH), (iii) lack of flame figures (excluding Wells syndrome), (iv) aberrant immunophenotype (LCA+/CD3– large cells), and (v) definitive response to lymphoma‐directed therapy.
- Comparison: while eosinophilia in MF has been described without peripheral eosinophilia [5, 6, 7, 8, 9, 10] and rare cases with histiocytosis have been reported [11, 12, 13], the dual and pronounced infiltration seen here has not been previously documented. This represents a unique diagnostic scenario.

This study is limited by its single‐case nature, which restricts generalizability. Besides, the diagnosis was made without T‐cell receptor clonality assessment due to unavailable technical facilities, relying instead on a comprehensive clinicopathological correlation and the compelling evidence of treatment response. Future studies should explore the immunologic pathways contributing to this phenomenon and assess whether similar cases exist in larger cohorts.

## 4 Conclusion

This case underscores that extensive eosinophilic and histiocytic infiltrates in a patient with known MF can represent a dramatic reactive phenomenon that obscures the underlying lymphoma. The presence of eosinophilic or histiocytic infiltration in MF poses significant diagnostic challenges, often leading to misdiagnosis or delayed diagnosis. Clinicians should consider MF as a potential differential diagnosis in cases with unexplained eosinophilia or LCH‐like histiocytic infiltration, even when initial histopathologic findings are inconclusive. Repeated biopsies, comprehensive IHC analysis, and TCR‐PCR studies are critical tools for differentiating reactive inflammatory infiltrates from true histiocytic neoplasms such as LCH.

## Author Contributions

Conceptualization: I.E. and S.M.V. Data curation: A.G. and K.K.H. Methodology: S.M., K.K.H., and A.G. Investigation: S.M.V. and S.M. Supervision: I.E. Validation: E.P., S.B., and S.M.V. Visualization: S.M., A.G., and K.K.H. Writing – original draft preparation: S.M.V., S.M., S.B., and I.E. Writing – review and editing: S.M.V., S.M., E.P., and I.E.

## Funding

The authors have nothing to report.

## Consent

Written informed consent was obtained from the patient to publish this report in accordance with the journal's patient consent policy.

## Conflicts of Interest

The authors declare no conflicts of interest.

## Supporting information


**Data S1:** cnr270412‐sup‐0001‐Supinfo.docx.

## Data Availability

Data sharing not applicable to this article as no datasets were generated or analyzed during the current study.
