# Supplementary material for: Tumoral Stage of Mycosis Fungoides, Misdiagnosed With Wells Syndrome and Langerhans Cell Histiocytosis Histologically: A Challenging Case and Review of the Literature
Source: Cancer Rep (Hoboken). 2025 Nov 30;8(12):e70412. doi: 10.1002/cnr2.70412 (PMC12665118; doi:10.1002/cnr2.70412)
Supplement: Supplementary file 1 — Data S1: cnr270412‐sup‐0001‐Supinfo.docx. [file CNR2-8-e70412-s001.docx]

**
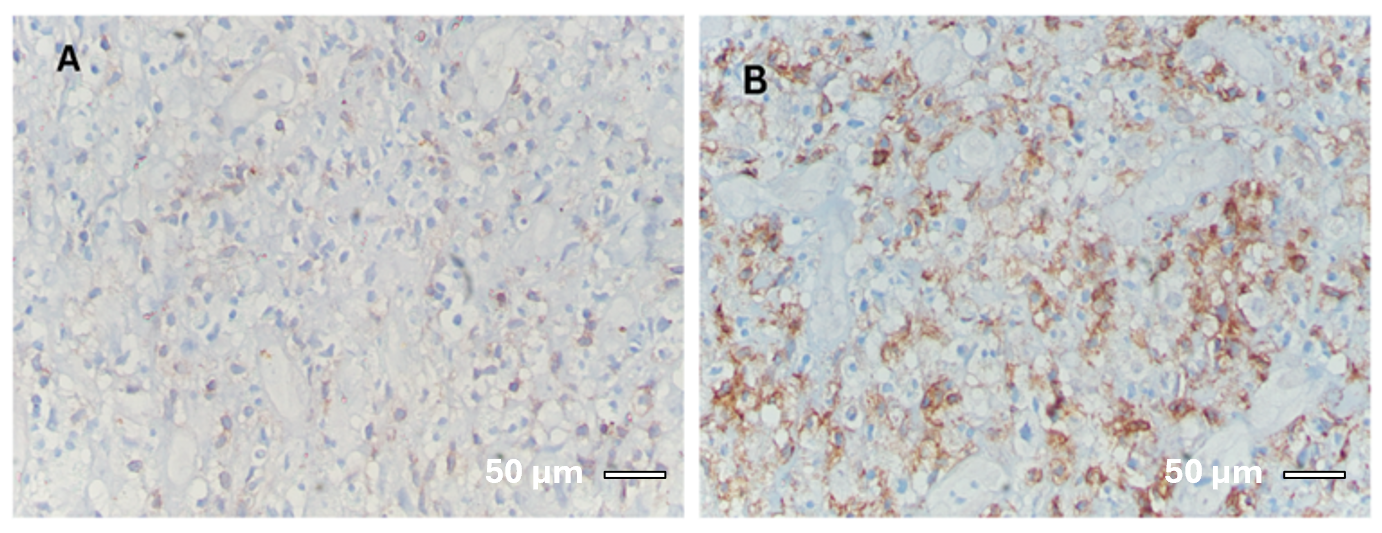
**

**Supplementary figure 1. Internal positive controls (third biopsy, corresponding to Figure 4).**

(A) CD3 positive in scattered small mature lymphocytes. (B) CD4 positive in a subset of background T-lymphocytes. Magnification: 400×. Scale bars: [50 µm]. Immunohistochemistry internal controls from the third biopsy sample (Figure 4). A. CD3 staining reveals positivity in scattered small, mature lymphocytes, serving as an internal positive control (400X). B. CD4 staining shows robust expression in a subset of background T-lymphocytes. (400X).
